# Supplementary material for: Patient prioritisation methods to shorten waiting times for elective surgery: A systematic review of how to improve access to surgery
Source: PLoS One. 2021 Aug 30;16(8):e0256578. doi: 10.1371/journal.pone.0256578 (PMC8404982; doi:10.1371/journal.pone.0256578)
Supplement: S1 Fig — (DOCX) [file pone.0256578.s002.docx]

PRISMA flow diagram for the major systematic review and displaying sub-review headings

Total citations from electronic database search

PubMed, EMBASE, SCOPUS, Web of Science, Cochrane

N = 7543

Identification


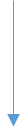

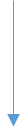


Excluded 2197 duplicate records

Deduplication


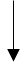


**Patient prioritisation in wait list n = 17**

Referral methods n = 9

Resource Management n = 5

Outsource to private sector n = 4

Reduce surgery cancellations n = 37

Sub-reviews (*n denotes cumulative number)

Wait time target n = 3

Perioperative time management n = 20

Accurate and manipulate wait lists n = 3

Continuous process improvement n = 5

Excluded 4984 irrelevant records

Excluded 166 irrelevant records

Potentially relevant citations from Title and Abstract screening N = 362

Total citations after electronic deduplication

N = 5346

Excluded 105 simulation/modelling studies

Eligible citations after assessing against inclusion and exclusion criteria N = 91

Relevant citations from

N = 196

Inclusion

Eligibility

Screening
